# Using Item Response Theory for the Development of a New Short Form of the Eysenck Personality Questionnaire-Revised

**DOI:** 10.3389/fpsyg.2018.01834

**Published:** 2018-10-02

**Authors:** Daiana Colledani, Pasquale Anselmi, Egidio Robusto

**Affiliations:** Department of Philosophy, Sociology, Education and Applied Psychology, School of Psychology, University of Padova, Padova, Italy

**Keywords:** short Eysenck personality questionnaire-revised, item response theory, 2PL, ESEM, DIF

## Abstract

The present work aims at developing a new version of the short form of the Eysenck Personality Questionnaire-Revised, which includes Psychoticism, Extraversion, Neuroticism, and Lie scales (48 items, 12 per scale). The work consists of two studies. In the first one, an item response theory model was estimated on the responses of 590 individuals to the full-length version of the questionnaire (100 items). The analyses allowed the selection of 48 items well discriminating and distributed along the latent continuum of each trait, and without misfit and differential item functioning. In the second study, the functioning of the new form of the questionnaire was evaluated in a different sample of 300 individuals. Results of the two studies show that reliability of the four scales is better than, or equal to that of the original forms. The new version outperforms the original one in approximating scores of the full-length questionnaire. Moreover, convergent validity coefficients and relations with clinical constructs were consistent with literature.

## Introduction

In the view of Eysenck (see Eysenck and Eysenck, 1975, 1991), the structure of personality may be effectively described by three main traits: psychoticism (P), extraversion (E), and neuroticism (N). These dimensions are also known as the “Giants Three” and represent basic, independent, and biologically founded traits. They characterize all subjects, with varying degrees, and allow for effectively describing behavioral, emotional, and individual differences among adults and young people. According to the authors, PEN traits do not represent pathological dimensions in themselves, but could lead to the development of abnormal conditions only in particular situations (Eysenck and Eysenck, 1991). In this perspective, neurosis and psychosis should be conceived as pathological exaggerations of the underlying traits of neuroticism and psychoticism (Eysenck and Eysenck, 1991; Mor, 2010).

Extraversion and neuroticism have been the first two dimensions included in the Eysenck's model and were conceptualized as orthogonal continua (Eysenck and Eysenck, 1964, 1991). The neuroticism dimension describes a trait opposed to emotional stability, and defines the degree to which a person is predisposed to experience negative affect (Eysenck and Eysenck, 1964, 1991; Mor, 2010). Individuals with high levels of this trait tend to be worried, apprehensive, moody, fed-up, and irritable (Eysenck and Eysenck, 1991; Eysenck and Barrett, 2013). Extraversion is the second dimension included in the model and depicts sociable, carefree, friendly, convivial, easygoing, and impulsive individuals. This trait is opposed to introversion which, in contrast, defines individuals introspective, quiet, serious, and reserved (Eysenck and Eysenck, 1975, 1991; Eysenck and Barrett, 2013). The third dimension included in the Eysenck's model has been psychoticism, or toughmindedness. The typical toughminded is an individual hostile, aggressive, untrusting, cold, unemotional, rude, lacking in human feelings, and unfriendly. On the opposite pole of the continuum, there are individuals with well-adjusted personality, agreeable, empathic, tolerant, conscientious, open-minded, friendly, and warm (Eysenck and Eysenck, 1975, 1991; Eysenck and Barrett, 2013).

Over the years, a series of instruments has been developed for the assessment of PEN traits on both young and adult people (e.g., Eysenck and Eysenck, 1964, 1975; Eysenck et al., 1985). These instruments also included a Lie (L) scale, which measures dissimulation and the tendency to deceive (Eysenck and Eysenck, 1964). Several contributions have been offered for the refinement of the psychometric properties of Eysenck's questionnaires, as well as for the development of brief versions (Eysenck et al., 1985; Francis and Pearson, 1988; Corulla, 1990; Francis et al., 1992; Francis, 1996). The psychometric properties and factor structure of all these instruments have been investigated in cross cultural research (e.g., Hosokawa and Ohyama, 1993; Maltby and Talley, 1998; Forrest et al., 2000; Qian et al., 2000; Scholte and De Bruyn, 2001; Aluja et al., 2003; Alexopoulos and Kalaitzidis, 2004; Dazzi et al., 2004; Francis et al., 2006; Karanci et al., 2006; Tiwari et al., 2009; Picconi et al., 2018). Unidimensionality of N and L scales has been widely supported in literature (e.g., Lajunen and Scherler, 1999; Ferrando, 2001; Ferrando and Chico, 2001; Ferrando and Anguiano-Carrasco, 2009; Dazzi, 2011). Contrasting results have been found concerning E scale: There are several studies supporting the unidimensionality of this scale (e.g., Rocklin and Revelle, 1981; Ferrando and Chico, 2001; Dazzi, 2011), but there is also some evidence suggesting the presence of two dimensions (Eysenck and Eysenck, 1963; Vidotto et al., 2008). Finally, there is large agreement in the literature that P scale comprises different facets (e.g., Howarth, 1986; Roger and Morris, 1991), which nevertheless contribute to a unique dimension (Chico and Ferrando, 1995; Dazzi, 2011).

Eysenck's instruments have been extensively employed for clinical, forensic, educational, and organizational purposes (e.g., Nyborg, 1997; Judge et al., 2000; Wood and Newton, 2003; Laidra et al., 2007; Smillie et al., 2009; Almiro et al., 2016), and all scales showed significant relations with a variety of psychologically and clinically relevant constructs and behaviors. Research, for instance, suggests that individuals with high levels of neuroticism may experience symptoms of anxiety and depression (e.g., Eysenck, 1991; Saklofske et al., 1995; del Barrio et al., 1997; Dazzi et al., 2004; Jylhä and Isometsä, 2006), and may also be more likely exposed to stress and health problems (e.g., Denney and Frisch, 1981; Huang et al., 2015; Bergomi et al., 2017). In contrast, extraversion appears to be mainly linked to adaptive social behavior, mental well-being, happiness, and life satisfaction (e.g., Lu, 1995; Mor, 2010; Gale et al., 2013). Moreover, this trait has been found to be negatively related to symptoms of anxiety and depression, to self-reported mental disorder and to health care use for psychiatric reasons (e.g., del Barrio et al., 1997; Jylhä and Isometsä, 2006). Finally, psychoticism has been often cited in relation to inappropriate social behaviors, such as unsafe sexual habits, heavy drinking, criminal behavior, dysfunctional impulsivity, gambling, and drug abuse (e.g., Barnes et al., 1984; Blaszczynski et al., 1985; Bogaert, 1993; Lodhi and Thakur, 1993; Francis, 1996; Conrad et al., 1997; Grau and Ortet, 1999; Hoyle et al., 2000; Chico et al., 2003; Heaven et al., 2004; Gudgeon et al., 2005; Colledani, 2018).

The short form of the Eysenck Personality Questionnaire-Revised (EPQ-R; Eysenck et al., 1985; Eysenck and Eysenck, 1991) includes 48 items (out of 100 of the EPQ-R), 12 per each of the four dimensions. This version of the instrument has been translated in several languages and is widely used, across different countries, for scientific and clinical purposes (Hosokawa and Ohyama, 1993; Aluja et al., 2003; Alexopoulos and Kalaitzidis, 2004; Dazzi et al., 2004; Francis et al., 2006; Tiwari et al., 2009; Sanavio et al., 2013). However, it suffers from the same drawbacks of the full-length version. In particular, P scale exhibited poor reliability with a restricted range of scores and a strong positive skewness (Bishop, 1977; Block, 1977; Claridge, 1981; Hosokawa and Ohyama, 1993; Katz and Francis, 2000; Alexopoulos and Kalaitzidis, 2004). In addition, several items showed differential item functioning (DIF) across gender (Eysenck et al., 1985; Eysenck and Eysenck, 1991; Lynn and Martin, 1997; Forrest et al., 2000; Karanci et al., 2006; Escorial and Navas, 2007), which makes the comparison between groups questionable.

A better selection of the items from the full-length version of the instrument could allow for reducing some of the aforementioned drawbacks. The present work aims at developing a new version of the short form of the EPQ-R with improved psychometric properties.

Item response theory (IRT; Bock, 1997; Thissen and Steinberg, 2009) is one of the most promising approaches to this aim. There are several successful applications of IRT for the development and validation of measurement scales (see, Da Dalt et al., 2013, 2015; Balsamo et al., 2014; Anselmi et al., 2015; Zanon et al., 2016; Sotgiu et al., 2018). Moreover, compared with classical test theory, IRT was found to provide more diagnostic information useful for the development of brief scales (Spence et al., 2012; Bortolotti et al., 2013; Petrillo et al., 2015). IRT allows for identifying the items that are best at discriminating different levels of the latent trait of interest, while ensuring that the entire trait continuum is covered. Selecting these items can result in a brief version of the scale that produces scores very similar to those obtained with the full-length scale and has the same external validity (i.e., the same correlations with other constructs; Reise and Henson, 2000; Spence et al., 2012). Moreover, IRT allows for detecting items that are unclear, ambiguous, or which exhibit DIF. These items should be not included in the brief scale. Despite advantages offered by IRT, only a few studies employed this approach for the refinement of Eysenck's instruments (e.g., Ferrando, 2001; Ferrando and Chico, 2001; Escorial and Navas, 2007; Maij-de Meij et al., 2008). Recently, Colledani et al. (2018) used IRT for developing a new version of the abbreviated form of the Junior EPQ-R (6 items per scale). The new version outperformed the original one on several aspects.

This work includes two main studies. In Study 1, a series of analyses were performed on the responses to the full-length version of the EPQ-R in order to select the 48 items (12 per each scale) with the best psychometric properties. In Study 2, the functioning of the new short form was tested in a new data sample. Reliability, validity and factor structure were examined. Relationships of the new scales with social desirability, the dimensions of the Five Factor Model (FFM), and clinically relevant constructs were verified.

## Study 1

### Participants

A total of 590 participants took part in the study (mean age = 36.69 years, *SD* = 14.16; from 18 to 75 years; 55.8% females). They were recruited from different Italian regions through convenience sampling. All participants were native Italian speakers and completed the questionnaire anonymously and voluntarily. All standards for research with human subjects were respected. Written informed consent was obtained from the participants. The project has been approved, now as later, by the Ethical Committee for the Psychological Research of the University of Padova since a prospective ethics approval was not required at the time when the research was conducted (Protocol n. 2622).

### Instruments

The participants were presented with the Italian version of the EPQ-R (Dazzi et al., 2004; Dazzi, 2011). The instrument consists of 100 dichotomous items (yes/no), 32 for P scale (e.g., “Should people always respect the law?,” “Do you enjoy hurting people you love?”), 23 for E scale (e.g., “Do you enjoy meeting new people?,” “Can you get a party going?”), 24 for N scale (e.g., “Would you call yourself a nervous person,” “Are you often troubled about feelings of guilt?”), and 21 for L scale (e.g., “Are all your habits good and desirable ones?,” “Have you ever cheated at a game?”). Administration of the questionnaire was individual and paper-and-pencil.

The Italian version of the questionnaire has good reliability and the four-factor structure was confirmed (α = 0.67, 0.78, 0.85, and 0.75 for P, E, N, and L scales, respectively; Dazzi et al., 2004; Dazzi, 2011). The reliability found in the current sample (α = 0.60, 0.79, 0.85, and 0.77 for P, E, N, and L scales) is in line with literature.

Studies in the Italian context aimed also to test the factor structure and the psychometric characteristics of the short version of the instrument (Dazzi et al., 2004). Consistently with cross-cultural findings, results supported the four-factor structure of the instrument and showed reliability coefficients satisfactory for E, N, and L scales, while lower for P (α = 0.37, 0.77, 0.83, and 0.70 for P, E, N, and L, respectively; Dazzi et al., 2004). The reliability found in the current sample (α = 0.40, 0.73, 0.83, and 0.73 for P, E, N, and L scales) is in line with literature.

### Analysis strategy

The two-parameter logistic (2PL) model (see Thissen and Steinberg, 2009) was separately estimated on the responses to each of the four scales of the questionnaire. This model describes the probability that a subject endorses a certain item as a function of the latent trait level of the subject (parameter θ), the “endorsability” level of the item (i.e., the ease of providing a “yes” response to that item; parameter ε), and the capability of the item in differentiating subjects with different trait levels (parameter δ). In the case of the P scale, for instance, the greater the value of parameter θ, the greater the level of psychoticism of the subject; the greater the value of parameter ε, the greater the ease of responding “yes” to the item (i.e., of providing a response that is indicative of the presence of psychoticism); the greater the value of parameter δ, the greater the capability of the item in differentiating between subjects with different levels of psychoticism. All the analyses were run using the packages “difR” (Magis et al., 2016) and “ltm” (Rizopoulos, 2012) for the statistical environment R (R Core Team, 2016).

The 2PL assumes unidimensionality of the scales. Confirmatory factor analyses were run on the data of each of the four scales (for a reasonable fit, CFI ≥0.90, RMSEA < 0.08; see Hu and Bentler, 1999; Marsh et al., 2004; Brown, 2006). These analyses confirmed the unidimensionality of N [$\chi_{\left(252\right)}^{2}$ = 1046.791, *p* ≤ 0.001; RMSEA = 0.073; CFI = 0.919] and L [$\chi_{\left(189\right)}^{2}$ = 532.901, *p* ≤ 0.001; RMSEA = 0.056; CFI = 0.900]. Fit indices of E scale were close to acceptance [$\chi_{\left(230\right)}^{2}$ = 808.417, *p* ≤ 0.001; RMSEA = 0.065; CFI = 0.890]. The unidimensional model did not fit the data of P scale [$\chi_{\left(464\right)}^{2}$ = 1841.233, *p* ≤ 0.001; RMSEA = 0.071; CFI = 0.467]. An exploratory factor analysis on this scale suggests a four-factor solution with 7 items out of 32 exhibiting cross-loadings. In line with literature (e.g., Howarth, 1986; Roger and Morris, 1991; Chico and Ferrando, 1995; Dazzi, 2011), this result confirms that P scale defines a complex and multifaceted construct.

#### Item selection for the new short scales

DIF and item fit statistics were used to identify the items with the poorest psychometric properties that were not included in the new short scales.

Three item fit statistics were used: infit, outfit (Wright and Masters, 1982), and the index suggested by Bock (1972). Infit and outfit are two χ^2^-based statistics, the former being effective in detecting unexpected responses to items close to a subject's trait level, the latter being effective in detecting unexpected responses to items far from the subject's trait level. In this work, items with infit and/or outfit higher than 1.4 (Wright and Linacre, 1994) were considered misfitting and not included in the new short scales. The index suggested by Bock involves grouping subjects into *n* categories on the basis of their latent trait level, and observed and expected proportions of subjects endorsing the item for each group are compared (Bock, 1972; Reise, 1990). In this work, subjects were grouped into four categories and the items which displayed a medium (0.3 ≤ Φ < 0.5) to large (Φ ≥ 0.5) effect size (Cohen, 1988) were not selected for inclusion in the new questionnaire.

Items exhibiting gender DIF were also excluded from the new questionnaire. Both uniform and non-uniform DIF were considered. The former is a systematic bias expressing a different probability of endorsing an item for the members of a specific group. The latter is a non-systematic bias which varies with the latent trait level. Females were used as reference group. Effect sizes of uniform and non-uniform DIF were evaluated by the *R*^2^ difference test (Nagelkerke, 1991; Gómez-Benito et al., 2009), with values higher than 0.035 denoting moderate DIF and values higher than 0.07 denoting strong DIF (Jodoin and Gierl, 2001; Magis et al., 2016).

Parameters ε and δ were examined to select, among the remaining items, those that allow for covering the entire trait continuum and with the greatest discrimination level.

#### Assessment of the psychometric characteristics of the new short scales

Reliability and validity of the newly developed PEN-L scales were evaluated and compared with those of the original short scales. Reliability was evaluated through Cronbach's α and test information function (TIF). TIF tells us how well the test measures the latent trait levels over the entire range of interest (Baker, 2001; Petrillo et al., 2015). The larger the value of TIF, the greater the accuracy with which the latent trait levels are measured. TIF depends on the latent trait range under consideration and on the number of items in the test (Baker, 2001). In this work, the old and new short scales had the same length (12 items), and TIF was defined on the same range of latent trait levels (−5 to 5). Validity was evaluated using a bias index and the correlation between scores obtained with full-length and short scales. The bias index was computed as the average difference (in absolute terms) between the parameters θ estimated on the full-length scales and those estimated on the short scales. Low biases suggest that the latent trait estimates obtained with the short scales approximate those of the full-length versions. In addition, the correlations between scores obtained with the full-length and short scales were computed and corrected for common items using the Levy's (1967) method.

### Results

Three of the 32 items of P scale exhibited uniform and non-uniform gender DIF of moderate (Items 68 and 91) or strong (Item 12) size. Fit statistics were adequate for all the items. From the remaining 29 items, 12 were selected taking into account their parameters ε and δ. This resulted in a new short scale, that differed from the original one for eight items (see Table 1). Specifically, Item 91 was changed because it showed uniform and non-uniform gender DIF of moderate size. These modifications allowed for obtaining a new scale with increased reliability (α increased from 0.40 to 0.62; TIF increased from 8.13 to 12.86) and with scores that better approximate those obtained with the full-length scale (bias decreased from 0.37 to 0.18, corrected correlation increased from 0.47 to 0.52). It is worth noting that Cronbach's α of the new 12-item scale (0.62) largely resembles that of the full 32-item scale (0.60).

**Table 1:** Easiness (ε) and discrimination (δ) parameters for the 32 items of the Psychoticism scale.

**New form**	**Original form**	**Item n**.	**Text**	**ε**	**δ**
		99	Would you feel very sorry for an animal caught in a trap?	−11.873	−0.121
		56	Do most things taste the same to you?	−10.789	−0.195
	✓	79	Do you try not to be rude to people?	−7.722	−0.354
		95	Do people tell you a lot of lies?	−2.538	−0.200
✓	✓	29	Do you prefer to go your own way rather than act by the rules?	−0.855	0.939
		9	Do you give money to charities?	−0.395	0.373
✓	✓	88	Is it better to follow society's rules than go your own way?	−0.045	1.247
✓		42	Have you often gone against your parents' wishes?	0.466	0.703
	✓	5	Do you take much notice of what people think?	0.502	0.150
	✓	75	Do you think people spend too much time safeguarding their future with savings and insurance?	0.713	0.313
✓		18	Should people always respect the law?	0.750	1.260
✓		64	Do you like to arrive at appointments in plenty of time?	0.953	0.846
✓		21	Are good manners very important?	0.993	1.580
✓	✓	41	Do good manners and cleanliness matter much to you?	1.439	1.763
		2	Do you stop to think things over before doing anything?	1.448	0.569
✓		81	Do you generally ‘look before you leap’?	1.807	0.708
✓		96	Do you believe one has special duties to one's family?	2.404	1.249
✓	✓	25	Would you take drugs which may have strange or dangerous effects?	2.725	1.138
	✓	48	Do you think marriage is old-fashioned and should be done away with?	3.343	0.552
		34	Do you have enemies who want to harm you?	3.734	0.367
✓		30	Do you enjoy hurting people you love?	3.775	0.910
	✓	59	Does it worry you if you know there are mistakes in your work?	4.024	0.538
	✓	7	Would being in debt worry you?	4.028	0.446
		73	Are there several people who keep trying to avoid you?	4.119	0.596
		68	Is (or was) your mother a good woman?	4.774	0.527
		85	Can you on the whole trust people to tell the truth?	4.986	−0.128
		12	Would it upset you a lot to see a child or an animal suffer?	5.307	0.750
	✓	54	Do you enjoy co-operating with others?	5.517	0.446
		14	Would it upset you a lot to see a child or an animal suffer?	5.623	−0.044
	✓	91	Would you like other people to be afraid of you?	6.036	0.242
✓		37	Do you have many friends?	6.638	0.515
		50	Are you more easy-going about right and wrong than most people?	8.580	−0.089

The items are ordered by increasing easiness. The items included in the new and in the original short forms are marked by “✓.”

Regarding the 23 items of E scale, only Item 55 exhibited uniform gender DIF of moderate size and no item showed misfit. Selecting 12 items upon the basis of their parameters ε and δ, we obtained a new E scale that differed from the original one for three items (see Table 2). The differences in reliability and validity of the new and original scales were small in size, nevertheless in favor of the new version (α increased from 0.73 to 0.75; TIF increased from 16.62 to 16.83; bias decreased from 0.21 to 0.19; corrected correlation increased from 0.74 to 0.77).

**Table 2:** Easiness (ε) and discrimination (δ) parameters for the 23 items of the Extraversion scale.

**New form**	**Original form**	**Item n**.	**Text**	**ε**	**δ**
	✓	47	Are you mostly quiet when you are with other people?	−5.623	−0.395
✓	✓	20	Do you enjoy meeting new people?	−1.914	1.613
	✓	11	Are you rather lively?	−1.605	1.311
✓	✓	58	Do you like mixing with people?	−1.592	2.182
		33	Do you prefer reading to meeting people?	−1.246	0.973
	✓	94	Do other people think of you as being very lively	−1.180	0.805
✓		28	Do you like going out a lot?	−1.105	1.193
		72	Do you often take on more activities than you have time for?	−0.832	0.570
		69	Do you often make decisions on the spur of the moment?	−0.755	0.489
		36	Do you have many friends?	−0.652	1.525
✓	✓	78	Can you get a party going?	−0.636	1.418
✓		67	Do you like doing things in which you have to act quickly?	−0.608	0.783
✓	✓	16	Can you usually let yourself go and enjoy yourself at a lively party?	−0.536	1.676
✓	✓	6	Are you a talkative person?	−0.468	1.211
		55	Do you like telling jokes and funny stories to your friends?	−0.365	0.705
		63	Do you nearly always have a ‘ready answer’ when people talk to you?	−0.230	0.401
✓	✓	45	Do you usually take the initiative in making new friends?	−0.171	1.741
✓	✓	24	Do you tend to keep in the background on social occasions?	0.151	1.658
✓	✓	51	Can you easily get some life into a rather dull party?	0.402	1.729
		40	Would you call yourself happy-go-lucky?	0.753	0.944
✓		61	Have people said that you sometimes act too rashly?	0.934	0.582
		1	Do you have many different hobbies?	0.974	0.519
✓	✓	90	Do you like plenty of bustle and excitement around you?	1.648	0.884

The items are ordered by increasing easiness. The items included in the new and in the original short forms are marked by “✓.”

Concerning N and L scales, no one item exhibited gender DIF or misfit. Therefore, items were selected considering their ε and δ parameters. For both scales, the new versions differed from the original ones for two items (see Tables 3, 4). Item 35 was present in the previous version of the N scale but it has not been included in the new one because of its redundant content. Reliability of the new scales largely resembles that of the original versions (α = 0.83, 0.82; TIF = 20.86, 20.80 for original and new N scale, respectively; α = 0.73, 0.74; TIF = 13.86, 14.15 for original and new L scale, respectively). Concerning N scale, a slight decrease of bias was observed (from 0.22 to 0.16). The other indexes remained substantially unchanged (bias = 0.20, 0.18 for original and new L scale, respectively; corrected correlation = 0.74, 0.75 for original and new L scale, respectively; 0.83, 0.84, for original and new N scale, respectively).

**Table 3:** Easiness (ε) and discrimination (δ) parameters for the 24 items of the Neuroticism scale.

**New form**	**Original form**	**Item n**.	**Text**	**ε**	**δ**
		87	Are you easily hurt when people find fault with you ot the work you do?	−1.652	0.930
		97	Are you touchy about some things?	−1.528	0.696
✓	✓	22	Are your feelings easily hurt?	−1.486	1.143
		92	Are you sometimes bubbling over with energy and sometimes very sluggish?	−1.186	1.025
✓	✓	80	Do you worry too long after an embarrassing experience?	−1.139	1.128
		13	Do you often worry about things you should not have done or said?	−1.027	1.084
		43	Do you worry about awful things that might happen?	−0.819	0.497
		74	Do you worry a lot about your looks?	−0.460	0.484
✓	✓	3	Does your mood often go up and down?	−0.319	1.785
		100	When your temper rises, do you find it difficult to control?	−0.164	0.848
✓	✓	31	Are you often troubled about feelings of guilt?	−0.114	1.445
✓		65	Have you often felt listless and tired for no reason?	−0.003	1.720
	✓	8	Do you ever feel “just miserable” for no reason?	0.007	1.392
✓	✓	17	Are you an irritable person?	0.225	1.527
	✓	35	Would you call yourself a nervous person?	0.250	2.582
✓	✓	46	Would you call yourself tense or “highly-strung”?	0.308	2.928
✓	✓	84	Do you often feel lonely?	0.308	1.669
✓	✓	83	Do you suffer from “nerves”?	0.394	2.833
✓	✓	26	Do you often feel “fed-up”?	0.591	1.713
		76	Have you ever wished that you were dead?	0.976	1.102
✓		70	Do you often feel life is very dull?	0.990	1.273
		60	Do you suffer from sleeplessness?	2.443	0.691
✓	✓	38	Are you a worrier?	2.948	0.721
		52	Do you worry about your health?	8.462	−0.132

The items are ordered by increasing easiness. The items included in the new and in the original short forms are marked by “✓.”

**Table 4:** Easiness (ε) and discrimination (δ) parameters for the 21 items of the Lie scale.

**New form**	**Original form**	**Item n**.	**Text**	**ε**	**δ**
		98	Are you always willing to admit it when you have made a mistake?	−4.705	0.427
	✓	57	As a child were you ever cheeky to your parents?	−2.153	0.536
		62	Do you always wash before a meal?	−2.120	0.614
✓		4	Have you ever taken the praise for something you knew someone else had really done?	−2.057	0.678
✓	✓	19	Have you ever blamed someone for doing something you knew was really your fault?	−1.687	0.869
✓	✓	71	Have you ever taken advantage of someone?	−0.909	1.638
✓		32	Do you sometimes talk about things you know nothing about?	−0.733	1.188
	✓	15	If you say you will do something, do you always keep your promise no matter how inconvenient it might be?	−0.693	0.898
✓	✓	66	Have you ever cheated at a game?	−0.508	1.183
✓	✓	44	Have you ever broken or lost something belonging to someone else?	−0.172	1.299
✓	✓	27	Have you ever taken anything (even a pin or button) that belonged to someone else?	−0.149	1.382
		49	Do you sometimes boast a little?	0.164	0.996
✓	✓	10	Were you ever greedy by helping yourself to more than your share of anything?	0.303	1.201
✓	✓	53	Have you ever said anything bad or nasty about anyone?	0.419	1.513
		77	Would you dodge paying taxes if you were sure you could never be found out?	0.421	0.473
✓	✓	86	Do you always practice what you preach?	0.423	1.052
		89	Have you ever been late for an appointment or work?	0.451	1.094
		39	As a child did you do as you were told immediately and without grumbling?	0.691	0.717
✓	✓	23	Are all your habits good and desirable ones?	0.712	1.080
✓	✓	93	Do you sometimes put off until tomorrow what you ought to do today?	1.155	1.212
		82	Have you ever insisted on having your own way?	5.265	0.173

The items are ordered by increasing easiness. The items included in the new and in the original short forms are marked by “✓.”

### Discussion

This study aimed at developing a new short version of the EPQ-R with improved psychometric characteristics. IRT based statistics allowed the identification of 48 items without gender DIF or misfit, well discriminating, and well distributed along the four latent traits continua. The new version of the P scale differs from the original one for eight items (out of 12), E scale for three, and N and L only for two. The largest improvement was reached for P scale, which in literature was found to perform less well than the other three scales (e.g., Bishop, 1977; Block, 1977; Claridge, 1981). In particular, the new version is not affected by gender DIF and outperforms the original one for reliability and approximation of the scores obtained with the full-length form. The new versions of the other three scales performed as well as, or slightly better than the original ones. Although small in size, these improvements are valuable taking into account that were obtained by substituting a small number of items and reducing content redundancy.

## Study 2

This study aimed at investigating the functioning of the new version of the short EPQ-R on a new data set. Other to reliability and factor structure, construct validity was evaluated by taking into account relationships with social desirability, the dimensions of the FFM, and measures of anxiety and depression.

### Participants

Participants were 300 native Italian speakers aged between 18 and 65 (mean age = 29.28, *SD* = 10.38; 60.2% females). They were recruited from different Italian regions using convenience sampling. All participants were presented with the new version of the short EPQ-R, whereas a subsample of 158 participants (mean age = 34.73, *SD* = 9.88; 68.7% females) also received the other measures. The participation to the study was anonymous and voluntary, and all standards for research with human subjects were respected. Written informed consent was obtained from the participants. The project has been approved, now as later, by the Ethical Committee for the Psychological Research of the University of Padova since a prospective ethics approval was not required at the time when the research was conducted (Protocol n. 2622).

### Instruments

The new form of the short EPQ-R devised in Study 1 was administered to all participants.

The five traits of the FFM of personality (i.e., extraversion, agreeableness, conscientiousness, emotional stability, and openness) were measured through the Italian version (Ubbiali et al., 2013; Chiorri et al., 2016) of the Big Five Inventory (BFI; John et al., 2008). The questionnaire consists of 44 items answered on a five-point Likert scale (from 1 “Strongly disagree” to 5 “Strongly agree”; e.g., “I see myself as someone who is full of energy” for extraversion; “I see myself as someone who is helpful and unselfish with others” for agreeableness; “I see myself as someone who perseveres until the task is finished” for conscientiousness; “I see myself as someone who worries a lot” for emotional stability; “I see myself as someone who is ingenious, a deep thinker” for openness). Convincing evidence was found concerning construct validity, factor structure, gender invariance, and reliability (α from 0.75 to 0.86; Ubbiali et al., 2013; Chiorri et al., 2016; α from 0.73 to 0.83 in the current sample).

The Impression Management (IM) scale of the Italian brief version (Bobbio and Manganelli, 2011) of the Balanced Inventory of Desirable Responding (BIDR; Paulhus, 1991) was also administered. The scale comprises 8 items answered on a six-point Likert scale (from 1 “Strongly disagree” to 6 “Strongly agree”) and assesses the conscious tendency of individuals to provide positively inflated self-descriptions (e.g., “I have never dropped litter on the street”). Internal consistency of the scale ranges from 0.73 to 0.81 (Bobbio and Manganelli, 2011; in the current sample, α = 0.75).

The trait scale of the State-Trait Anxiety Inventory (STAI-Y; Spielberger et al., 1983; Pedrabissi and Santinello, 1989) was used to evaluate anxiety. The scale comprises 20 items answered on a four-point Likert scale (from 1 “Not at all” to 4 “Very much”). The instrument evaluates the tendency of people to experience general anxiety and the relatively stable predisposition to view stressful situations as threatening (e.g., “I am regretful”). The Italian version of the questionnaire showed adequate validity and reliability (α from 0.85 and 0.90; Pedrabissi and Santinello, 1989; in the current sample, α = 0.92).

Finally, the Italian version of the Patient Health Questionnaire-9 (PHQ-9; Spitzer et al., 1999; Kroenke et al., 2001) was used to evaluate depressive symptoms. The questionnaire is a self-administered instrument and assesses the nine DSM-IV (American Psychiatric Association, 2000) criteria for depression. Respondents are asked to evaluate the presence of depressive symptoms over the last 2 weeks through nine items scored on a four-point Likert scale (from 0 “Not at all” to 3 “Nearly every day”; e.g., “Feeling tired or having little energy”). This instrument showed adequate reliability (α from 0.86 to 0.89), and good sensitivity and specificity (see Kroenke et al., 2001). In the current sample, α equals 0.81.

### Analysis strategy

Reliability of the new version of the short EPQ-R was tested through Cronbach's α. Construct validity was evaluated by computing convergent validity coefficients and by analyzing the factor structure of the instrument.

Convergent validity was evaluated considering correlations between the four PEN-L traits, the five dimensions of FFM, social desirability, and indexes of depression and trait anxiety. According with literature, L scores are expected to positively correlate with the IM scale of the BIDR (e.g., Gillings and Joseph, 1996), while PEN traits are expected to correlate with BFI scales, depression and trait anxiety. In particular, positive correlations are expected between E scores of the EPQ-R and the extraversion measure of the BFI, while negative correlations are expected between P scale and agreeableness and conscientiousness. Positive correlations are also expected between N scale of the EPQ-R and the neuroticism measure of the BFI (e.g., McCrae and Costa, 1985; Draycott and Kline, 1995; Saggino, 2000; Barbaranelli et al., 2003; Scholte and De Bruyn, 2004; Heaven et al., 2013). Neuroticism, in addition, is expected to positively correlate with indexes of anxiety and depression (STAI-Y; Spielberger et al., 1983; PHQ-9; Spitzer et al., 1999; Kroenke et al., 2001). In contrast, extraversion is expected to negatively correlate with these two clinical indexes.

An Exploratory Structural Equation Model (ESEM; Asparouhov and Muthén, 2009) was run to evaluate the factor structure. The ESEM framework represents an integration of confirmatory factor analysis (CFA), structural equation modeling (SEM), and exploratory factor analysis (EFA). ESEMs give access to all the common statistics of SEM/CFA but, at the same time, overcome the restrictions associated with the confirmatory approach. CFA fixes non-target loadings to zero and, therefore, it may be inadequate to handle complex and multifaceted constructs where many cross-loadings may be expected (Marsh et al., 2009, 2010, 2011, 2014). When this is the case, fit problems and upward-biased estimates of correlations between factors can be observed (Cole et al., 2007; Marsh and Hau, 2007; Marsh et al., 2010). As in EFA, ESEMs allow for the free estimation of cross-loadings between items and non-target factors. In this work, ESEM was run using Mplus7 (Muthén and Muthén, 2012), and the WLSMV as estimator (weighted least squares mean and variance-adjusted). This method is recommended for binary or ordinal observed data (e.g., Flora and Curran, 2004; Brown, 2006) such as the dichotomous items of the EPQ-R. In the model, the 48 items were the indicators and four factors were modeled. The GEOMIN oblique rotation was used. To evaluate the goodness of fit of the model, several fit indexes were considered: χ^2^, Comparative Fit Index (CFI; Bentler, 1990), Weighted Root Mean Square Residual (WRMR; Yu, 2002), and Root Mean Square Error of Approximation (RMSEA; Browne and Cudeck, 1993) with its 90% confidence interval (90% CI) and the test of close fit (CFit; Browne and Cudeck, 1993). A solution fits the data well when χ^2^ is non-significant (*p* ≥ 0.05). Since this statistic is sensitive to sample size, the other fit measures were also considered. In particular, a solution fits the data well when CFI is close to 0.95 (0.90 to 0.95 for reasonable fit), WRMR is close to 1.0, and RMSEA is smaller than 0.06 (0.06 to 0.08 for reasonable fit) with CFit non-significant (see Hu and Bentler, 1999; Marsh et al., 2004; Brown, 2006).

### Results

Cronbach's α coefficients were 0.55, 0.80, 0.81, and 0.70 for P, E, N, and L scales, respectively. These values were consistent with those of Study 1. Compared with the original version, the largest improvement was reached for P scale, as observed in Study 1.

Convergent validity coefficients are reported in Table 5. All the four PEN-L traits correlated in the expected direction with the considered constructs. E scale showed a strong positive relation with the extraversion measure of the BFI (0.727). P scale was negatively related to agreeableness (−0.323) and conscientiousness (−0.321). N scale was strongly correlated with neuroticism (0.709). Relations with anxiety and depression were also in the expected directions. N scale showed positive relations with scores of PHQ-9 (0.619) and STAI-Y (0.697), while moderate negative relations were found between these two indexes and E scale (*r* = −0.409, −0.405 for PHQ-9 and STAI-Y, respectively). Finally, L scale showed a strong positive correlation with the IM scale of the BIDR.

**Table 5:** Cronbach's αs and correlations between the four PEN-L traits, STAI-Y, PHQ-9, BIDR-IM, and the five BFI dimensions.

	**α**	**Psychoticism**	**Extraversion**	**Neuroticism**	**Lie**
STAI-Y	0.92	0.092	−0.409^***^	0.697^***^	−0.259^**^
PHQ-9	0.81	0.099	−0.405^***^	0.619^***^	−0.202^*^
BIDR-IM	0.75	−0.429^***^	0.195^*^	−0.293^***^	0.561^***^
BFI-Extraversion	0.79	0.058	0.727^***^	−0.369^***^	0.132
BFI-Agreeableness	0.73	−0.323^***^	0.317^***^	−0.241^**^	0.242^**^
BFI-Conscientiousness	0.83	−0.321^***^	0.220^**^	−0.385^***^	0.334^***^
BFI-Neuroticism	0.80	0.021	−0.363^***^	0.709^***^	−0.110
BFI-Openness	0.78	0.094	0.262^**^	−0.126	0.065

* *p < 0.05*,

** *p < 0.01*,

*** *p < 0.001*.

Results of the ESEM supported the four-factor structure of the instrument {$\chi_{\left(942\right)}^{2}$ = 1122.686, *p* < 0.001; RMSEA = 0.025 [0.019, 0.031]; CFit ≅ 1.000; CFI = 0.930; WRMR = 0.864}. The model is represented in Table 6. All items loaded on the intended factor and cross-loadings were, in general, lower than those observed on the target-factor.

**Table 6:** Exploratory structural equation modeling.

**Item**	**Psychoticism**	**Extraversion**	**Neuroticism**	**Lie**
29	0.279^**^	−0.058	0.303^**^	−0.089
88	0.317^***^	0.066	0.101	−0.123
96	0.444^***^	0.065	0.017	0.050
41	0.913^***^	−0.076	−0.058	0.032
25	0.384^**^	−0.193	−0.119	−0.551^***^
42	0.312^**^	0.045	0.049	−0.304
37	0.596^***^	0.084	0.141	−0.255
64	0.457^***^	0.118	0.051	0.117
21	0.728^***^	−0.148	−0.050	0.058
30	0.743^***^	−0.236	−0.352^*^	−0.001
81	0.546^***^	0.223	0.286^**^	0.004
18	0.531^***^	−0.075	−0.063	−0.342^**^
16	−0.065	0.796^***^	−0.152	−0.141
78	0.083	0.482^***^	0.037	0.151
20	−0.081	0.775^***^	−0.067	0.176
51	0.182	0.605^***^	−0.014	0.014
67	0.075	0.317^***^	−0.052	−0.027
6	0.090	0.374^***^	−0.060	0.030
28	0.180	0.678^***^	0.033	0.154
58	−0.077	0.865^***^	−0.201	0.060
90	−0.004	0.732^***^	−0.017	−0.242
45	0.100	0.600^***^	0.020	0.100
24	−0.067	0.817^***^	−0.164	−0.094
61	0.396^***^	0.499^***^	0.414^***^	−0.038
65	−0.034	−0.162	0.540^***^	−0.184^*^
17	0.065	0.136	0.701^***^	0.187^*^
46	0.024	0.019	0.844^***^	0.245^**^
3	0.045	0.176	0.798^***^	−0.007
38	−0.217^*^	−0.151	0.658^***^	−0.016
80	−0.262^*^	−0.296^*^	0.313^***^	−0.040
26	−0.201^*^	−0.131	0.683^***^	−0.107
70	0.002	−0.191	0.619^***^	−0.070
31	−0.135	−0.140	0.605^***^	−0.029
22	−0.168	−0.114	0.479^***^	0.199^*^
38	−0.156	−0.210	0.558^***^	0.048
83	0.045	−0.034	0.910^***^	0.323
66	−0.094	−0.089	0.011	0.664^***^
27	−0.385^*^	0.047	−0.109	0.318^**^
32	0.018	−0.319^**^	−0.374^***^	0.235^*^
86	0.110	−0.057	−0.243^**^	0.365^***^
4	−0.402^**^	−0.005	−0.049	0.483^***^
93	0.000	−0.301^**^	−0.201^*^	0.449^***^
23	−0.144	0.069	−0.167	0.369^***^
53	0.060	0.043	−0.107	0.639^***^
10	0.054	0.050	−0.011	0.566^***^
44	−0.290^*^	−0.113	−0.066	0.502^***^
71	−0.359^***^	0.060	0.026	0.476^***^
19	−0.302^**^	−0.054	−0.326^**^	0.191^*^
**CORRELATION WITH**				
Extraversion	0.043			
Neuroticism	0.04	−0.198^***^		
Lie	−0.125	0.081	−0.258^**^	

*Standardized factor loadings and factor correlations (N = 300)*.

* *p < 0.05*,

** *p < 0.01*,

*** *p < 0.001. Bolded coefficients are target loadings*.

### Discussion

The analyses performed in this study provide further evidence concerning the adequate psychometric properties of the new short form of the EPQ-R. Concerning reliability, results are in line with those of Study 1 and confirm that, compared with the original version, the largest improvement was observed for P scale. Concerning validity, both the factor structure of the instrument and its convergent validity are supported.

## Final remarks

This work aimed at developing a new and improved version of the short form of the EPQ-R. This instrument is well-known and widely used in different settings. However, some weaknesses have been pointed out, especially for P scale (e.g., Bishop, 1977; Block, 1977; Claridge, 1981). IRT approach was used to develop the new instrument. This approach allowed for removing items with misfit or gender DIF, and for identifying items that were best at discriminating different levels of traits, while ensuring that the respective continua were covered. As suggested in literature, following these criteria for item selection should lead to a short scale with the same psychometric properties of the full-length instrument (Reise and Henson, 2000; Spence et al., 2012). In fact, results of this work show that the new short form of the EPQ-R approximated the scores obtained with the full-length form better than the original short version. In addition, convergent validity of the new scale was consistent with literature (e.g., Saklofske et al., 1995; Gillings and Joseph, 1996; del Barrio et al., 1997; Dazzi et al., 2004; Jylhä and Isometsä, 2006; Mor, 2010). The moderate to strong relationships between Eysenck's traits and clinical constructs provide further evidence toward the usefulness of assessing these traits in clinical settings.

A strength of the present work is that it provides a solution to some well-known drawbacks of the full-length EPQ-R and of its short form existing in the literature (Eysenck et al., 1985; Eysenck and Eysenck, 1991). The largest improvement was obtained for P scale. The new version is not affected by gender DIF and outperforms the original one for reliability and approximation of the full-length form. The new versions of the other three scales performed as well as the original ones, or slightly better. These improvements are small in size, yet notable considering that were obtained by substituting a small number of items and reducing content redundancy.

In the present work, separate analyses have been performed on each of the four scales by using a unidimensional IRT model. An alternative could have been examining the four scales at once through a multidimensional IRT (MIRT) model (see Haberman et al., 2008; Reckase, 2009). MIRT models offer some advantages over unidimensional IRT models. They could allow for better understanding the traits measured by an instrument and how well individual items measure each of them (Ackerman, 1994). Moreover, MIRT models could provide a more precise estimation of scale reliability (Cheng et al., 2009) and item parameters (Finch, 2010). In the present work, some of these advantages are not very relevant. On the one hand, the factor structure of the EPQ-R has been widely tested and validated in the literature (e.g., Hosokawa and Ohyama, 1993; Maltby and Talley, 1998; Forrest et al., 2000; Qian et al., 2000; Scholte and De Bruyn, 2001; Aluja et al., 2003; Alexopoulos and Kalaitzidis, 2004; Dazzi et al., 2004; Francis et al., 2006; Karanci et al., 2006; Tiwari et al., 2009; Picconi et al., 2018). On the other hand, for scales whose length is analogous to that of the four EPQ-R scales (i.e., from 21 to 32 items), the unidimensional IRT models have been found to provide item parameter estimates whose precision exceeds or equals that of the estimates produced by the MIRT models (Finch, 2010). Finch (2010) investigated the precision of MIRT estimates on tests measuring a number of traits as small as two. For larger numbers of traits (e.g., the four traits of the EPQ-R), the number of parameters of a MIRT model increases considerably. Thus, the sample size of Study 1 (590 individuals) could have not been appropriate for performing a multidimensional analysis.

Concerning P scale, despite notable improvements, reliability remains rather low. This result, however, was expected. P scale, in fact, maybe because of its complex and clinical nature, is the most problematic and controversial of the instrument (e.g., Eysenck et al., 1985). Future research, therefore, should try to develop a new pool of items effective in capturing the multifaced aspects of this trait.

In the present work, a new short version of the EPQ-R has been devised, which consists of 12 items per each of the four scales. An abbreviated form exists also in literature (Francis et al., 1992) that consists of only 6 items per scale. This abbreviated form suffers of the same weaknesses that have been pointed out for the other Eysenck's questionnaires. Future research should try to devise a new version of the abbreviated form by using the IRT approach.

## Data availability statement

The raw data supporting the conclusions of this manuscript will be made available by the authors, without undue reservation, to any qualified researcher.

## Author contributions

DC contributed to the conception and design of the study, conducted the research, performed the statistical analyses, and wrote the first draft of the manuscript. DC and PA wrote sections of the manuscript. All authors contributed to manuscript revision, read and approved the submitted version.

### Conflict of interest statement

The authors declare that the research was conducted in the absence of any commercial or financial relationships that could be construed as a potential conflict of interest.
